# Transformative Dental Care in Pediatric Moebius Syndrome: Bridging Oral Health and Systemic Management During Prolonged Hospitalization

**DOI:** 10.1111/scd.70031

**Published:** 2025-06-17

**Authors:** Renata Zoraida Rizental Delgado, Marcus Bueno, Leda Mugayar, Juliana Bertoldi Franco

**Affiliations:** ^1^ Inclusive Care Clinic College of Dentistry University of Illinois Chicago Illinois USA; ^2^ Department of Dentistry Central Institute Clinical Hospital of Medical School of the University of São Paulo São Paulo São Paulo Brazil; ^3^ Department of Dentistry Children and Adolescent Institute Clinical Hospital of Medical School of the University of São Paulo São Paulo São Paulo Brazil

**Keywords:** dental care, disabled health care, hospital dentistry, moebius syndrome

## Abstract

Moebius syndrome is a rare congenital disorder of unknown etiology. It is characterized by non‐progressive paralysis of the facial and abducens cranial nerves due to atrophy of their nuclei, often accompanied by malformations in the upper and lower limbs. This report aims to highlight the critical role of hospital dentistry in managing a 6‐year‐old patient with Moebius syndrome. The patient was on mechanical ventilation via tracheostomy following a cardiorespiratory arrest at birth and has been hospitalized in a long‐term care institution. Feeding and medication were administered via gastrostomy, and she was under a regimen of phenobarbital, phenytoin, and benzodiazepines for epilepsy control. Dental follow‐up, initiated at birth, included monthly bed visits and medical team consultations. Key interventions included extraction of deciduous teeth to prevent bronchopulmonary aspiration, management of sialorrhea using scopolamine and botulinum toxin, selective wear of deciduous canines to mitigate self‐mutilation, and monitoring drug‐induced gingival hyperplasia. This case underscores the essential role of dental care in maintaining oral health, preventing complications such as respiratory infections, and enhancing the quality of life for syndromic patients under prolonged mechanical ventilation.

## 1 Introduction

Hospital dentistry is vital to comprehensive care for patients with complex medical, physical, or developmental conditions. It integrates oral health into broader patient care, addressing challenges such as hygiene maintenance in intubated patients, preventing secondary infections, and mitigating complications arising from systemic conditions [1].

Moebius syndrome, first described in 1880 by Alfred Graefe and later named after Paul Julius Moebius in 1888, is extremely rare, with an estimated prevalence between 1 in 50 000 and 1 in 500 000 live births. Patients with rare congenital disorders, such as Moebius syndrome, present unique challenges that underscore the importance of hospital dentistry. This syndrome is characterized by congenital paralysis of the facial and abducens cranial nerves, leading to significant functional and aesthetic impairments. These include difficulties in swallowing, chewing, and speaking, alongside the inability to express emotions through facial movements. These deficiencies result in facial paralysis, strabismus, and orofacial abnormalities [2, 3, 4].

The syndrome's etiology remains multifactorial, involving genetic, environmental, and potentially ischemic factors, and its presentation is highly variable, necessitating individualized care strategies. These disruptions can be caused by genetic factors, mutations in specific genes, poorly controlled diabetes, hyperthermia, maternal exposure to infections, and the use of substances such as misoprostol, alcohol, cocaine, thalidomide, and benzodiazepines, among others [2, 3, 5, 6].

The syndrome's systemic effects may necessitate hospitalization, especially when respiratory, renal, cardiac, or feeding issues are present. Patients with Moebius syndrome may depend on mechanical ventilation or gastrostomy feeding, further complicating oral health management [3, 5, 7].

Craniofacial malformations are usually present, such as facial asymmetry, eyelid ptosis, convergent strabismus, hypertelorism, epicanthal folds, deafness, microstomia, micrognathia, hypoglossia, aglossia, or ankyloglossia [5, 7]. In addition to oral malformations, musculoskeletal deficiencies can also occur, such as limb deformities, especially in the lower feet, pectoral aplasia, extremity defects, heart problems, and respiratory dysfunctions [8, 9].

The most common oral manifestations associated with Moebius syndrome are periodontal diseases, dental hypoplasia, sialorrhea, lack of lip sealing, lingual fissures, decreased facial and lingual muscle tone, micrognathia, microstomia, bifid uvula, cleft palate, open bite, and early childhood dental caries. Thus, the presence of the dental surgeon within the multidisciplinary team involved in meeting the needs of these patients becomes essential [2, 3, 4, 5, 6, 7].

The oral health challenges in patients with Moebius syndrome are profound, including issues like sialorrhea, gingival hyperplasia, and risks associated with mechanical ventilation, such as oral candidiasis and pneumonia. The literature emphasizes the importance of a multidisciplinary approach, combining efforts from dentists, physicians, speech therapists, and caregivers to manage these complications effectively [10, 11].

The treatment of Moebius syndrome is symptomatic since the disease, although not progressive, has no cure. A multidisciplinary approach involving speech therapists (treatment of sialorrhea and perioral musculature), physical therapists (motor and respiratory therapy), physicians (medical care and prescriptions), psychologists (family counseling), nurses (hygiene care, medication administration, and general nursing support), and dentists (for oral care, preventive measures, and dental procedures) is essential to preserving both the physical and mental capacities of these patients. This approach should be initiated as early as possible. Within their realities, patients with Moebius syndrome have a life expectation equivalent to that of people without disabilities since no vital organ is affected by the syndrome [10, 11, 12, 13, 14].

Emerging treatments, including botulinum toxin (BTX) injections and anticholinergic agents, have demonstrated efficacy in managing sialorrhea, a prevalent concern in these patients. Furthermore, studies underline the need for consistent oral hygiene protocols to prevent secondary infections and improve overall health outcomes in hospitalized syndromic patients. Saliva production is stimulated by the parasympathetic nervous system. For the treatment of sialorrhea, sympathomimetic drugs such as scopolamine or BTX infiltration into the minor salivary glands can be used. These agents block the production of acetylcholine in the synaptic cleft, reducing saliva production [1, 11, 12].

The role of the dental surgeon within multidisciplinary teams caring for hospitalized patients with Moebius syndrome cannot be overstated. From addressing oral hygiene to implementing preventive and therapeutic strategies, dental professionals contribute significantly to reducing risks such as aspiration pneumonia and improving patients’ quality of life. This case report highlights hospital dentistry's critical contributions in managing a pediatric patient with Moebius syndrome, emphasizing the need for an interdisciplinary approach to achieve optimal outcomes.

### 1.1 Case Report

**FIGURE 1:**
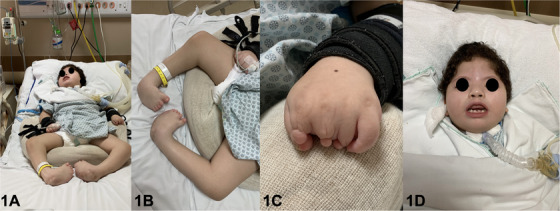
(A) Patient in a PICU presenting multiple physical deformities, non‐verbal, non‐responsive, and fully dependent on care; (B) Lower limb deformities; (C) Deformities in hand; (D) Extra‐oral frontal view observing microcephaly, palpebral ptosis, and little facial expression.

This report describes a 6‐year‐old girl diagnosed with Moebius syndrome who has been hospitalized in the Pediatric Intensive Care Unit (PICU) of a Public University Hospital in Suzano, São Paulo, Brazil (Auxiliary Hospital of Suzano) (Figure 1A) since birth due to a cardiorespiratory arrest, requiring mechanical ventilation via tracheostomy. The patient is nonverbal, unresponsive, and entirely dependent on general care. Feeding and medication were administered via gastrostomy. The patient exhibited classic features of Moebius Syndrome, including limb and hand deformities (Figure 1B,C), microcephaly, palpebral ptosis, and limited facial expression (Figure 1D). She had seizure episodes controlled with the use of phenobarbital, phenytoin, and benzodiazepines, which contributed to mild drug‐induced gingival hyperplasia, controlled with oral hygiene and biofilm management.

**FIGURE 2:**
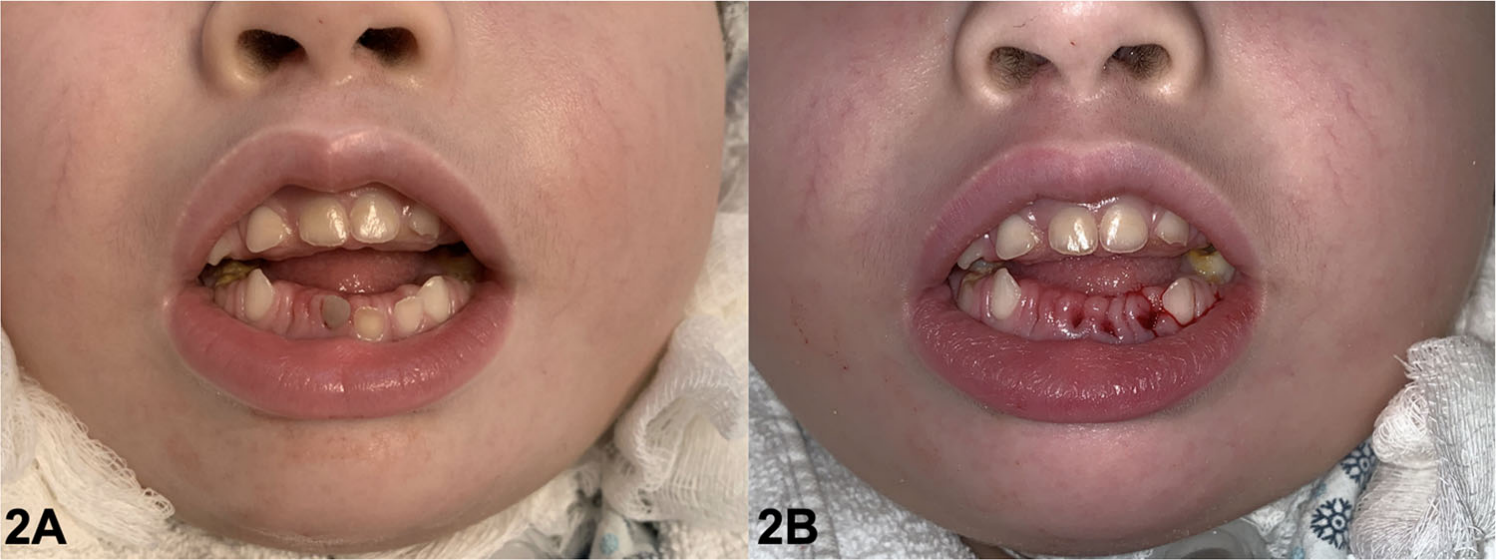
(A) Frontal intraoral view observing anterior open bite and lower right central incisor with color change due to internal resorption; (B) Frontal view observing the alveoli after the extraction of deciduous teeth that presented mobility and risk of aspiration.

Dental follow‐up commenced at birth with monthly visits, and daily oral care was provided by trained nursing staff using a sponge oral swab and an essential oil‐based mouth antiseptic. Over 6 years of follow‐up, the deciduous teeth were extracted to prevent bronchopulmonary aspiration, particularly during the exfoliation phase, as evidenced clinically by grade III mobility and the change in crown color indicating internal resorption (Figure 2A and B).

**FIGURE 3:**
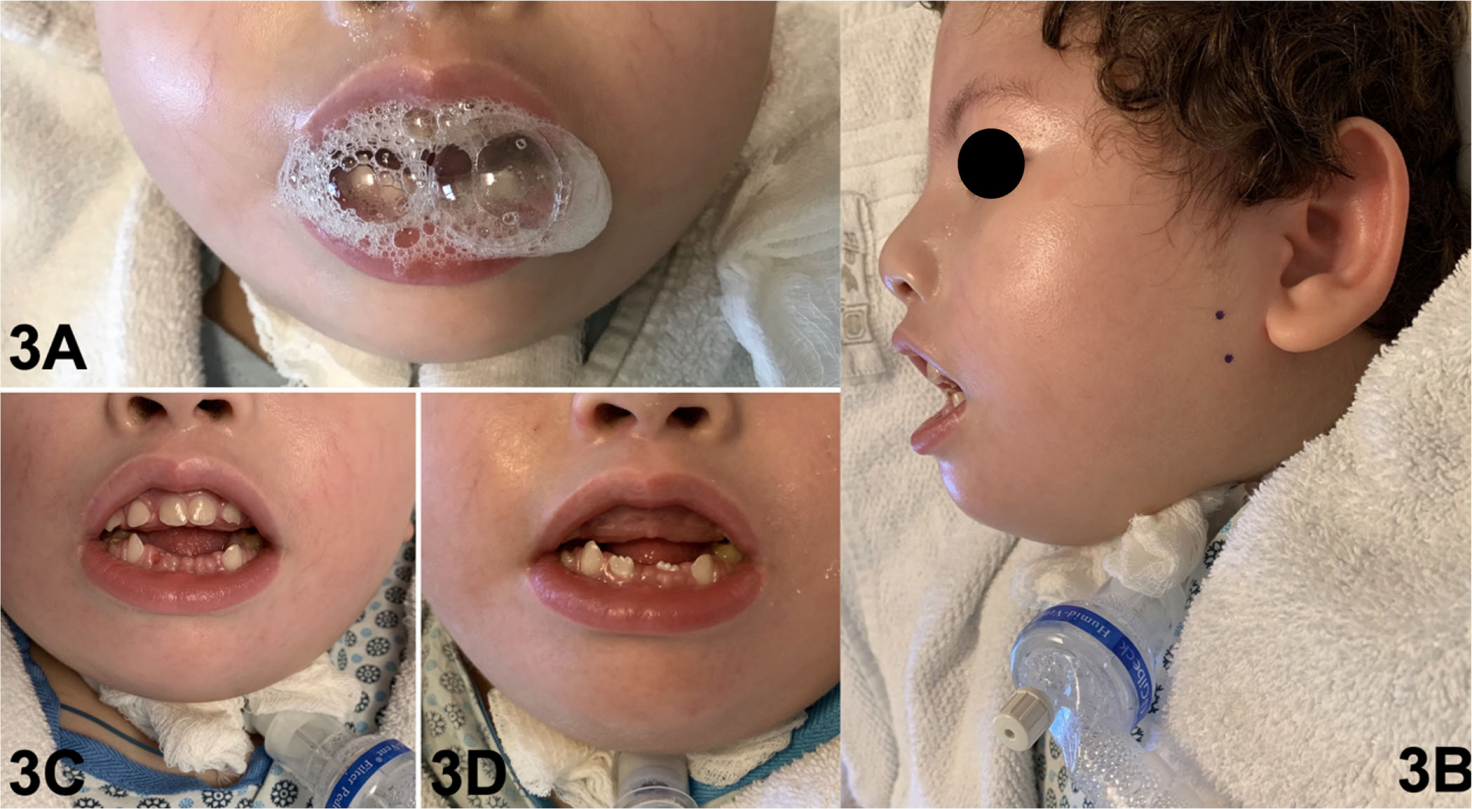
(A) Front view observing severe sialorrhea; (B) BTX‐A application points in parotid; (C) 2‐month follow‐up of BTX‐A application; (D) 4‐month follow‐up of BTX‐A application demonstrating the effectiveness of the treatment.

Another characteristic present in the patient is sialorrhea, which requires professional control. This objective was achieved using drying measures (scopolamine via gastrostomy for 3 years—one drop per kilogram of body weight administered two–three times a day) [1]. Scopolamine was administered via gastrostomy during the first years of life due to its low cost and the patient's good clinical response. However, from the fourth year of life onward, scopolamine ceased to produce satisfactory results, primarily due to intestinal constipation. Botulinum toxin‐A (BTX‐A) was applied, guided by anatomical features, to the major salivary glands (10U in the bilateral parotid gland and 15 U in the bilateral submandibular gland from 4 years old, administered twice a year) [12] as a treatment for sialorrhea, yielding effective results that lasted for 6 months without the need for additional anticholinergic medications (Figure 3A–D).

**FIGURE 4:**
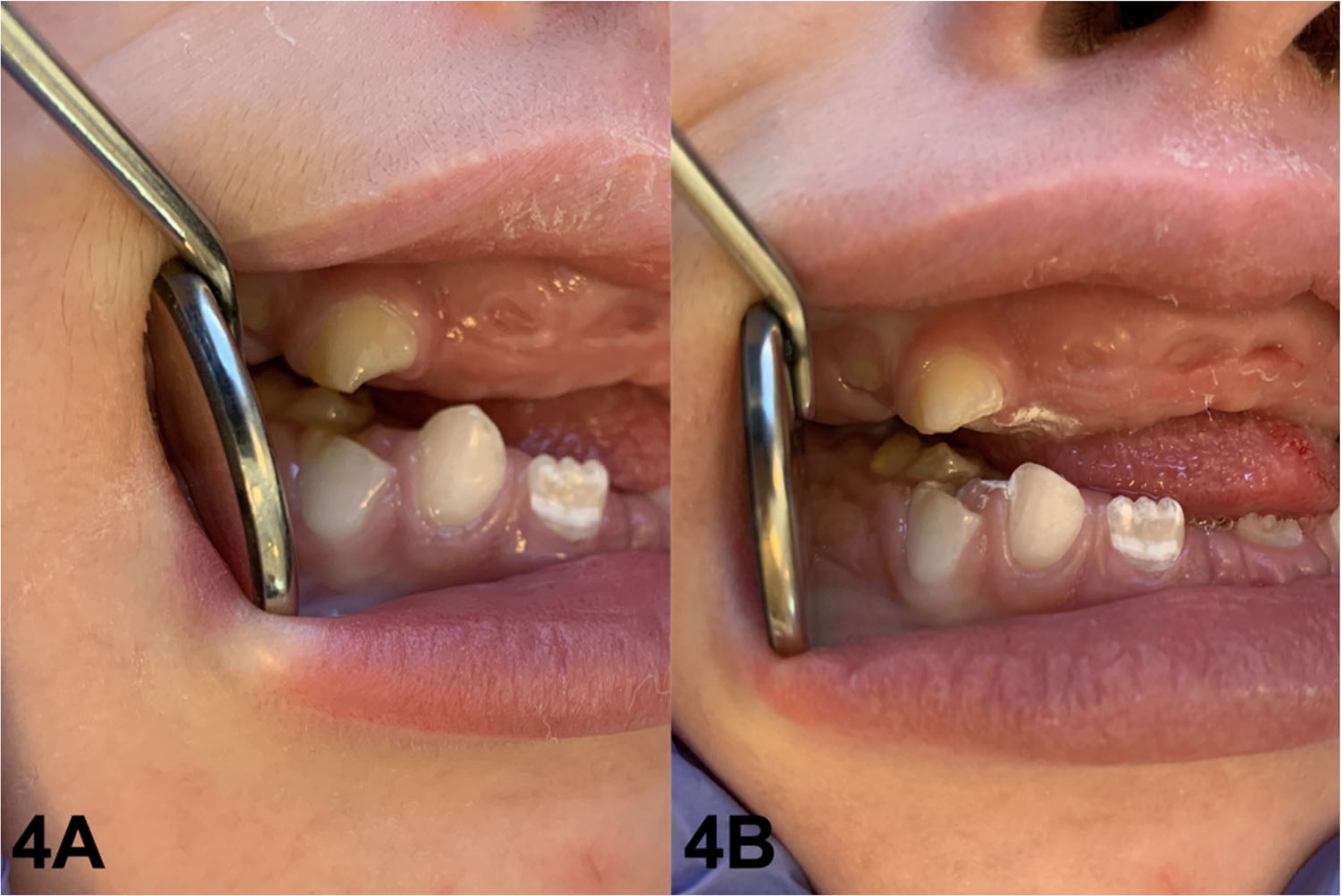
(A) Upper and lower right deciduous canines before selective wear; (B) Upper and lower right deciduous canines after selective wear.

Self‐mutilation of the lower lip and hands, caused by the pointed anatomy of the deciduous teeth, was a significant concern for the team. The patient presented with pain and bleeding in the oral mucosa as a result of the self‐inflicted injuries. To address this issue, selective reshaping of the upper teeth was performed to reduce the risk of injury, alleviate discomfort, and enhance the patient's safety and well‐being (Figure 4A and B).

Dental treatment strategies for the situations encountered during the patient's hospitalization:

Preventing aspiration risks: Extraction of mobile deciduous teeth to eliminate the risk of aspiration.

Managing sialorrhea: Use of scopolamine and BTX‐A injections to reduce severe sialorrhea and its complications.

Preventing self‐mutilation: Selective wear of deciduous canines to minimize injury.

Oral hygiene maintenance: Nursing staff provide oral daily care to prevent infections.

Monitoring drug‐induced effects: Regular monitoring and management of drug‐induced gingival hyperplasia by oral hygiene instructions and biofilm control.

## 2 Discussion

This case aligns with documented Moebius syndrome characteristics, such as facial asymmetry, eyelid ptosis, and facial paralysis [2, 3, 4, 5].

Dental care for syndromic patients, especially those on mechanical ventilation, is crucial to prevent complications like respiratory infections. The presence of a dentist on the multidisciplinary care team helps reduce risks such as aspiration pneumonia by addressing oral hygiene and managing complications like sialorrhea [13].

The lack of oral care in patients on mechanical ventilation contributes to the emergence of hospital infections, compromising the patient's quality of life, general health, and the evolution of their case. Thus, the presence of dentists in the multi professional team responsible for monitoring the reported case was important not only for specific care related to Moebius Syndrome but also for preventing respiratory tract contamination by opportunistic microorganisms [9, 13].

The clinical management of the presented case aligns with and extends upon findings reported in the scientific literature. Moebius syndrome is frequently associated with significant oral and systemic challenges, many of which were observed in this patient. One of the most prominent features, sialorrhea, was managed effectively using BTX‐A and anticholinergic agents. The literature consistently supports these interventions as minimally invasive and highly effective for controlling sialorrhea, as evidenced in studies by Heikel et al. [12] and Scully et al. [11]. These approaches improved the patient's quality of life and mitigated risks of aspiration pneumonia, aligning with published outcomes. The use of BTX‐A for sialorrhea control proved minimally invasive and effective, aligning with contemporary practices in managing sialorrhea [11, 12].

The extraction of mobile deciduous teeth to prevent bronchopulmonary aspiration reflects established best practices in hospital dentistry for mechanically ventilated patients. Literature by Oda et al. [13] underscores the critical nature of such interventions in reducing the risk of respiratory infections linked to oral microbial translocation. This case further highlights the need for comprehensive oral evaluations to proactively identify potential risks [13].

Syndromic patients on mechanical ventilation are at heightened risk of oral complications and systemic infections. Dental care, including tooth extraction and sialorrhea management, reduces the risk of aspiration pneumonia and improves overall health outcomes [1, 14].

Additionally, selective dental wear to avoid self‐mutilation illustrates an innovative adaptation of standard dental procedures. Although less frequently reported in the literature, this approach aligns with the principles of personalized care and trauma prevention for syndromic patients. The patient's tooth anatomy required tailored interventions to prevent complications, a practice supported by studies on customized dental care for individuals with craniofacial abnormalities [10].

Drug‐induced gingival hyperplasia, a known side effect of anticonvulsants, was consistently monitored and managed, preventing additional complications. Dental care significantly improves health outcomes and reduces the risk of secondary infections [1, 14].

Oral health is intrinsically linked to general health, particularly for hospitalized patients. Dental interventions can significantly influence recovery trajectories and quality of life [1, 14]

In the literature, previously published studies report the dental care of patients with Moebius syndrome being provided in dental clinics, without major systemic complications related to the syndrome [4, 7]. The presented clinical case exhibits great complexity due to the patient being hospitalized since birth as a result of sequelae from cardiorespiratory arrest, such as the need for mechanical ventilation and gastrostomy, cognitive impairments, dependence on care, and the necessity of multiple dental interventions. This case is considered a pioneering report on Moebius syndrome in a hospital setting.

This case underscores the critical role of hospital dentistry within multidisciplinary care of integrated dental professionals in ICU teams, facilitates the management of oral health issues, reduces systemic risks, and enhances patient outcomes. Furthermore, the case demonstrates the need for continuous education among healthcare professionals regarding the specific needs of patients with disabilities, as well as research to refine and expand interventions for complex syndromic conditions such as Moebius syndrome, particularly concerning the impact of preventive measures for maintaining oral health and improved treatments for sialorrhea.

## 3 Conclusion

This case highlights the indispensable role of hospital dentistry in managing syndromic patients, particularly those with complex oral and systemic health needs. Dental interventions, including tooth extraction, sialorrhea management, and self‐mutilation prevention, address specific dental challenges and contribute to overall health improvement and enhanced quality of life. Integrating dental professionals into multidisciplinary teams ensures comprehensive care and optimal outcomes for pediatric patients with Moebius syndrome.

## Ethics Statement

The authors have nothing to report.

## Conflicts of Interest

The authors declare no conflicts of interest.

## Data Availability

The data that support the findings of this study are available on request from the corresponding author. The data are not publicly available due to privacy or ethical restrictions.
